# Supplementary figures and images for: The lipid elongation enzyme ELOVL2 is a molecular regulator of aging in the retina
Source: Aging Cell. 2020 Jan 14;19(2):e13100. doi: 10.1111/acel.13100 (PMC6996962; doi:10.1111/acel.13100)

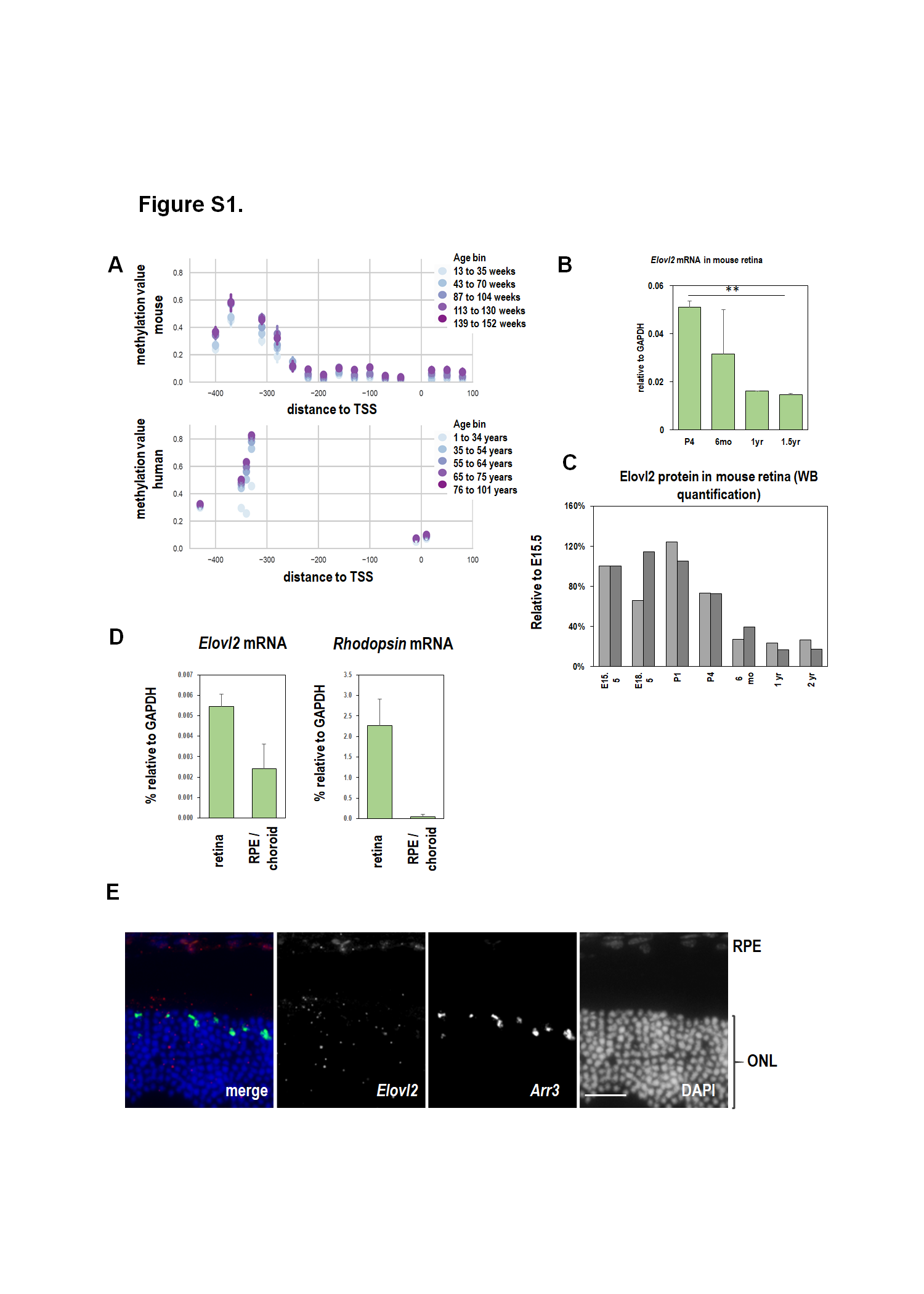

Supplement: Supplementary file 1 [file ACEL-19-e13100-s001.tif]

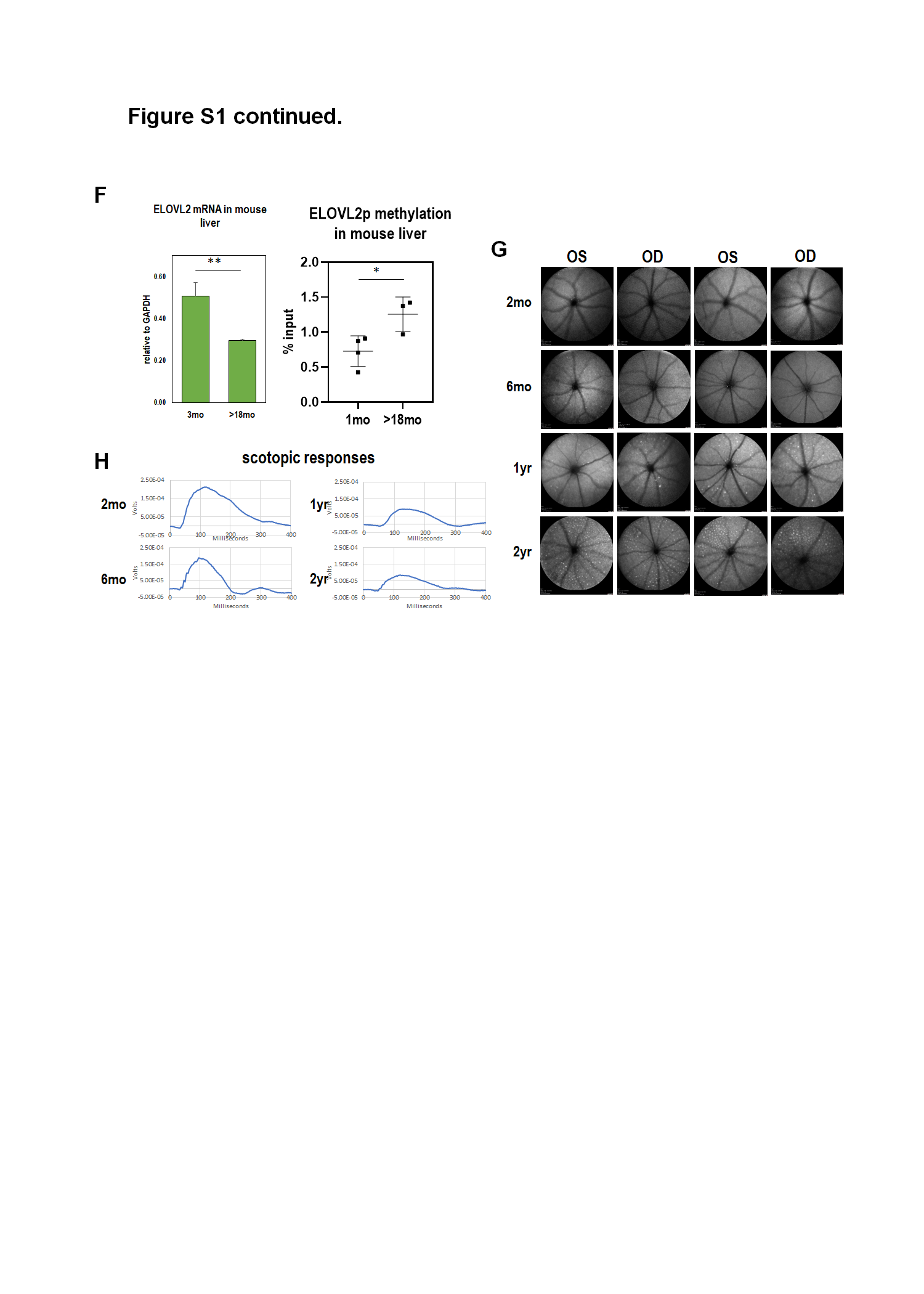

Supplement: Supplementary file 2 [file ACEL-19-e13100-s002.tif]

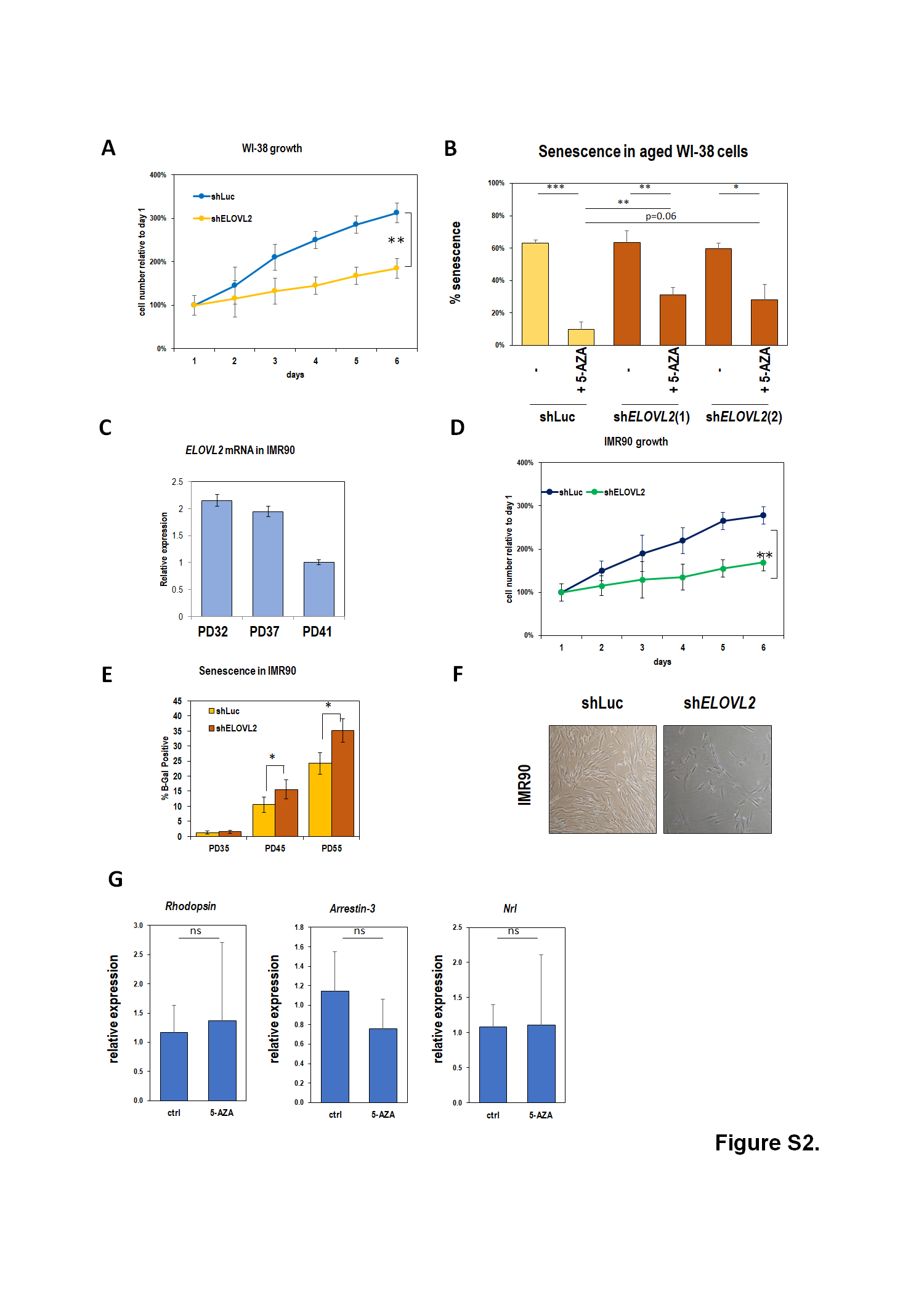

Supplement: Supplementary file 3 [file ACEL-19-e13100-s003.tif]

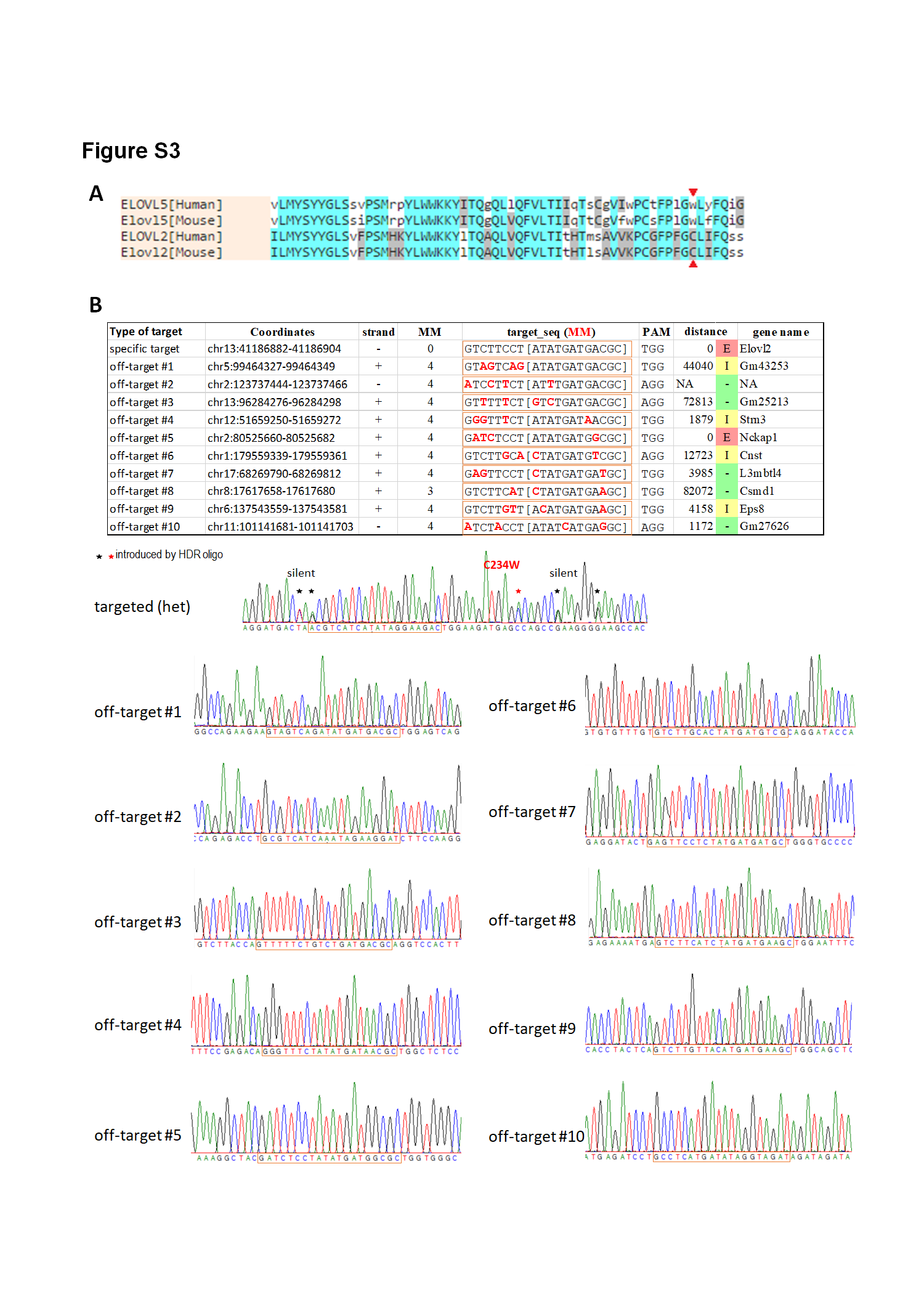

Supplement: Supplementary file 4 [file ACEL-19-e13100-s004.tif]

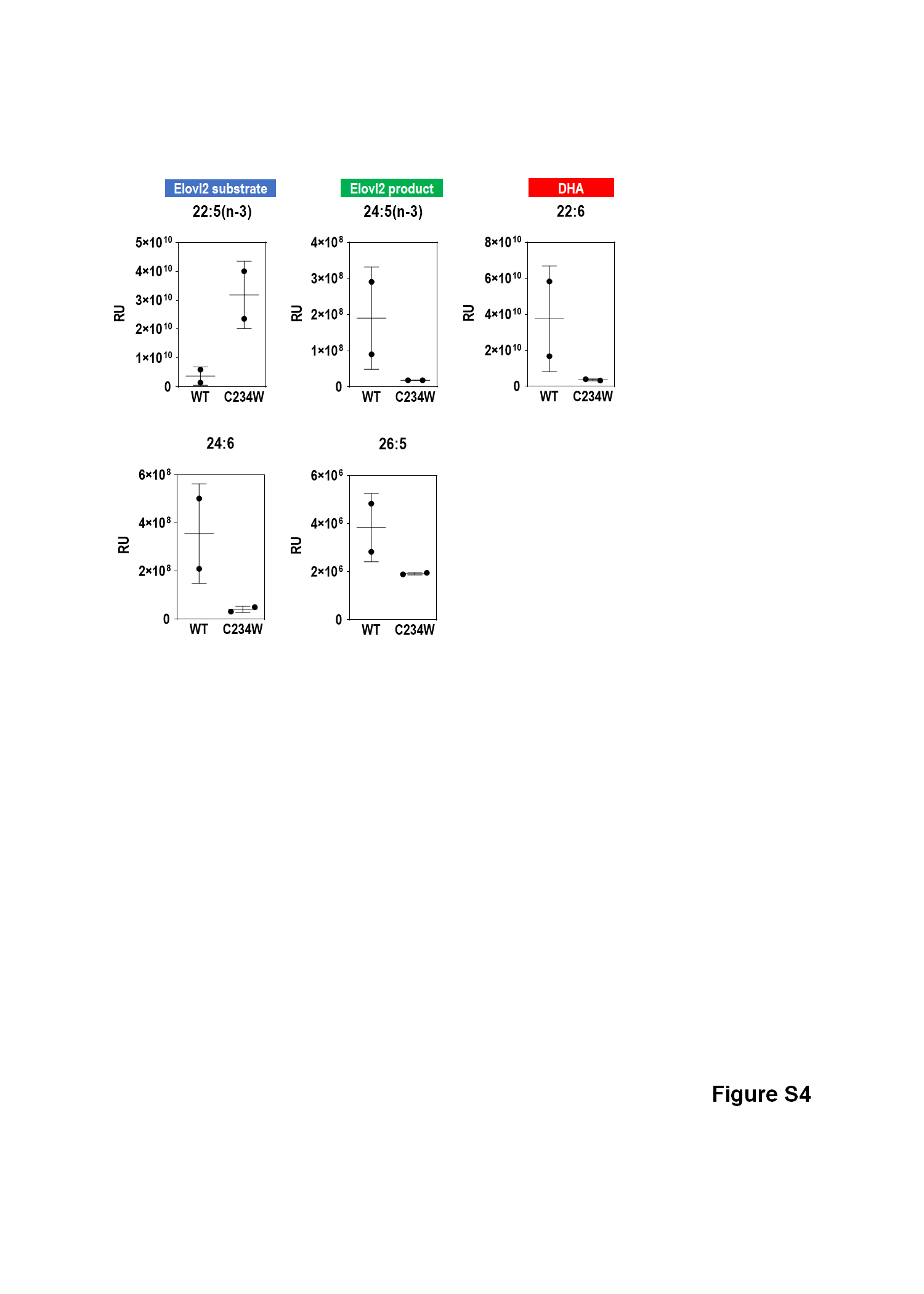

Supplement: Supplementary file 5 [file ACEL-19-e13100-s005.tif]

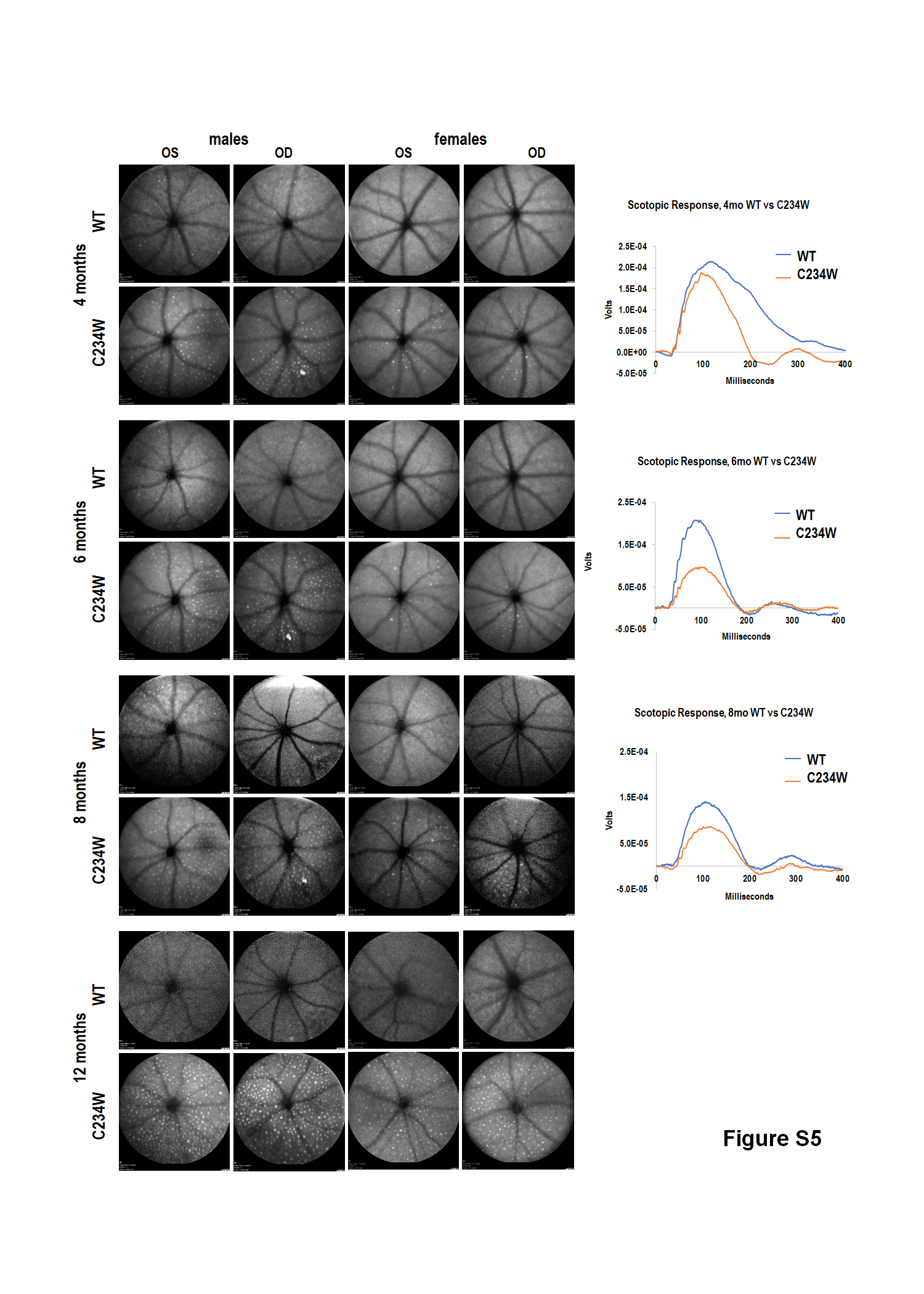

Supplement: Supplementary file 6 [file ACEL-19-e13100-s006.tif]

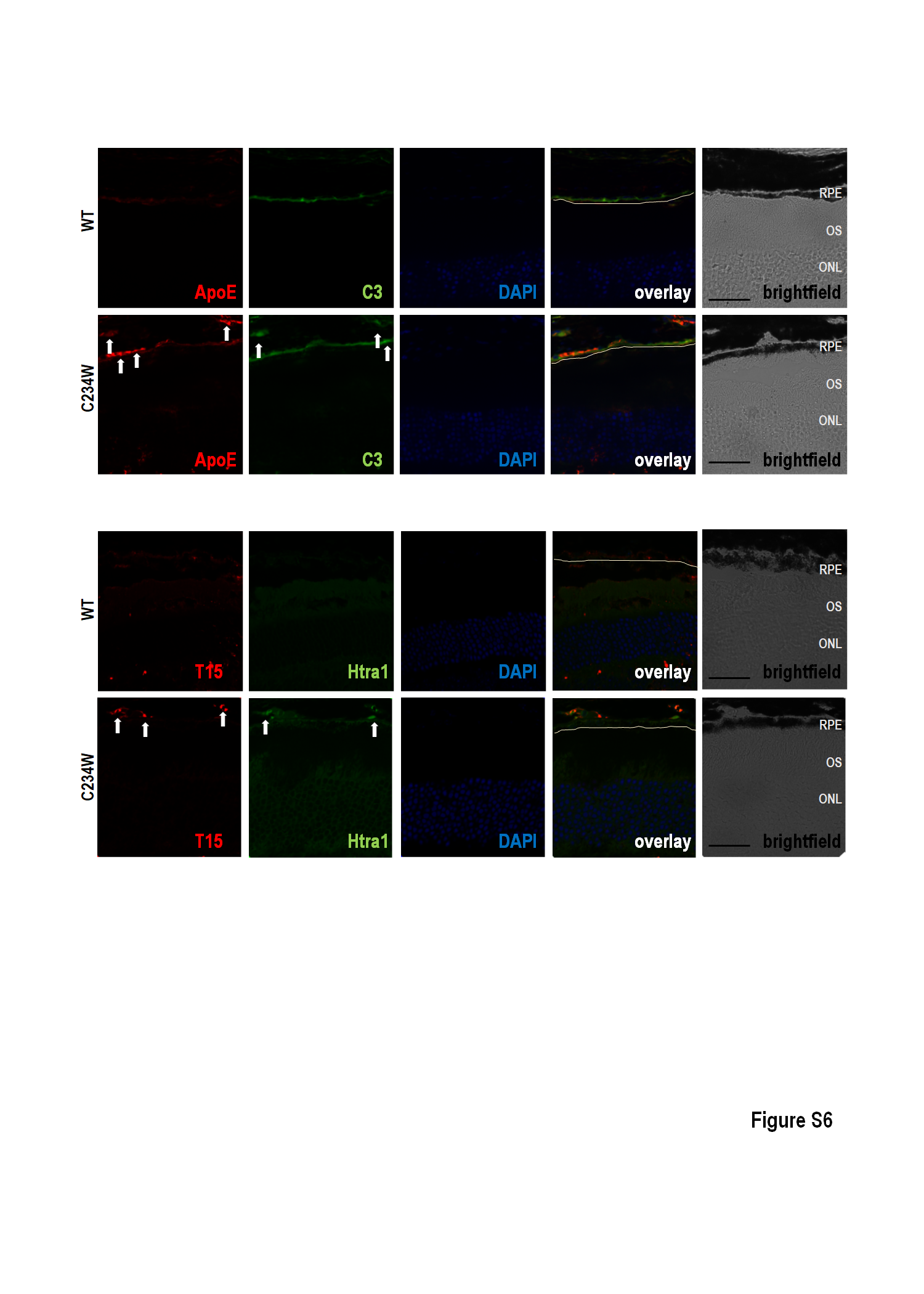

Supplement: Supplementary file 7 [file ACEL-19-e13100-s007.tif]
