# Supplementary material for: The lipid elongation enzyme ELOVL2 is a molecular regulator of aging in the retina
Source: Aging Cell. 2020 Jan 14;19(2):e13100. doi: 10.1111/acel.13100 (PMC6996962; doi:10.1111/acel.13100)
Supplement: Supplementary file 8 [file ACEL-19-e13100-s008.docx]

| **Off-targets** | **Sequence (5' -> 3')** | **Sequence (5' -> 3')** |
| --- | --- | --- |
| E2off-1 | ATTGCCTTATTAGGAGGAAAC | GAGAGTGATGAGCTTAATTTG |
| E2off-2 | GCTGGAAATTCACTGAAGAC | TTGGCAACACCAAAGAAC |
| E2off-3 | TCTGGAGGTACTGGTTAG | GAACACCTGCAGTCATAG |
| E2off-4 | GCCTCATTTACTACAGTAGTC | TCCATAGAGGAGTGAGAGTAAG |
| E2off-5 | CTCCCAAGTGTCGGGATTA | CTACTTCCCCAGCCCTTATAG |
| E2off-6 | CCAGCTATTGAGCGTGAAG | ACATTCCCTGAGTGCCTAC |
| E2off-7 | TCTATGAGGGTGCTGAGTC | CACCCAGGATCTTCATATAGG |
| E2off-8 | GACATTCTATTGGAGGGTTTAC | CTGCCTTGCTATATCTTTCTAC |
| E2off-9 | CCAAAGAGCATCACTAAGG | TCGGTTATGTCTTCGACTG |
| E2off-10 | GGAGGTCAGAAAGTCATTG | GAAGTCGATCACTGGAAAG |
| **MeDIP primers** |  |  |
| hELOVL2 prom. | CGATTTGCAGGTCCAGCCG | CAGCGGGTGGGTATTCCTG |
| mELOVL2 prom. | AGCTCCTCCGCTACTC | CCAGCCCTTGGTCATC |
| **qPCR primers** |  |  |
| mRhodopsin | ACCTGGATCATGGCGTTG | TCGTTGTTGACCTCAGGCTT |
| mOpsin | TGTACATGGTCAACAATCGGA | ACACCATCTCCAGAATGCAAG |
| mNrl | AGTCTCCAGGGAAGCTGTGC | TGGGACTGAGCAGAGAGAGG |
| mGAPDH | TCAACAGCAACTCCCACTCTTCCA | ACCCTGTTGCTGTAGCCGTATTCA |
| hELOVL2 | GCGGATCATGGAACATCTAA | CCAGCCATATTGAGAGCAGA |
| hACTB | CACCATTGGCAATGAGCGGTTC | AGGTCTTTGCGGATGTCCACGT |
| mRpe65 | ACCAGAAATTTGGAGGGAAAC | CCCTTCCATTCAGAGCTTCA |
| mRd8 | GGTGACCAATCTGTTGACAATCC | GCCCCATTTGCACACTGATGAC |
| Megamer | CAAAGCCCTTTCAAGCCAAGTCACGATTCTAATTTTAATTTGGCTAAGGAACACAGAAATAGGCCCAAACTCAAGTTGAACAAGGGCTTCTGACACTTTGATAACTTCGTATAGCATACATTATACGAAGTTATGATAGGCAGAGCATCAGTGCGAGAACAACGTTCTCAGGTTCTGGCAGGGTAGAGAAGCAGTAACTGCAACCTTGCACAGCTCTGGAAGGTCCACCTAACCAGTTCTCAAACTCAGATGCGTGCTGAGTGAAGCAAGCCAGAGTGCAGGCCACAGCCACTTACCTCCACCAGCATATACGCAGAAAGAAGTGTGATTGCGAGGTTATACAAGGTGAGGATGCCCCTGAGAGACAGAGCAGGCCTGTTCTTCATGTACTTGTTACCCAGCCATATCGAGAGCAGGTACGTGATGGTGAGGATGAAGGTGGGAAGGTAAGAGTCCAGCAGGAACCACCCGCGAACTCGAGAATCTGTAAAGAAATGCTTACGGTGAGGAGCCCAAGGAGGGATGTCCCTGTAATTAGCAACTCTAACACACATACTCATCTGTGCAACGGGCGTCCCCATCGATGGTGCACGCCCCACGATAACTTCGTATAGCATACATTATACGAAGTTATGTCTCCGTGAACACAAGCCCCGTCCTCACCGAGTACTCGGTGTTTGAGATGAACTCACATAAACAGGTCAAGTTGCTTTTCCCTGAACAAATTCTCAGAC | |

Table S1

| **Immunostaining** | **Company, Cat#** | **RRID** |
| --- | --- | --- |
| TEPC 15 | Sigma M1421 | AB_1163630 |
| HtrA | Santa Cruz sc-377050 | AB_2813838 |
| C3 | Santa Cruz sc-58926 | AB_1119819 |
| C5-b9 | Santa Cruz sc-66190 | AB_1119840 |
| ApoE | Santa Cruz sc-13521 | AB_626691 |
| **MeDIP** |  |  |
| 5-methylcytosine | Millipore MABE146 | AB_10863148 |
| **Western blot** |  |  |
| ELOVL2 | Santa Cruz sc-54874 | AB_2262364 |
| Histone H3 | Cell Signaling 9715 | AB_331563 |

Table S2
